# Growth in *Nephrops norvegicus* from a tag-recapture experiment

**DOI:** 10.1038/srep35143

**Published:** 2016-10-11

**Authors:** Paula S. Haynes, Patricia Browne, Liam Fullbrook, Conor T. Graham, Lee Hancox, Mark P. Johnson, Valentina Lauria, Anne Marie Power

**Affiliations:** 1 Ryan Institute, School of Natural Sciences, National University of Ireland Galway, Ireland; 2 Marine and Freshwater Research Centre, Department of Life Sciences, Galway-Mayo Institute of Technology, Galway, Ireland; 3 Marine Institute, Plymouth University, Plymouth, United Kingdom

## Abstract

*Nephrops norvegicus* is a commercially valuable fishery in the EU but management of stocks is challenging due to difficulties in aging individuals and calculating growth and biomass production. Growth of *N. norvegicus* was estimated by releasing 1177 tagged individuals in western Ireland in Summer 2013 and recapturing these in 2014 (n = 207, an average of 344 days later) and 2015 (n = 38, 654–665 days later). Moulting occurred twice per year in approximately half of the males and only once in females. Mean growth increments after approximately one year were 5.1 mm Carapace Length (CL) in males and 1.4 mm CL in females. After two years, males had grown by 12.0 mm CL and females by 4.6 mm CL, on average, across size classes. Low variation in growth increments was seen across female size classes, but significantly lower growth was observed in larger males, meeting an important assumption of the Von Bertalanffy Growth Function. Asymptotic carapace lengths were 70.8 mm (males) and 55.2 mm (females) with respective growth constants (k) of 0.161 yr^−1^ and 0.077 yr^−1^. The results suggest that this is a very productive fishery and that survivability of returns from creel fishing is high.

The Norway lobster, *Nephrops norvegicus*, is one of the most commercially important demersal species in the Northeast Atlantic, with increased landings over the past 50 years^1^,^2^. Stocks are managed via common European Union regulations (Common Fisheries Policy -CFP) and national stock assessments that are co-ordinated internationally at the EU’s Scientific, Technical and Economic Committee for Fisheries (STECF) and the International Council for Exploration of the Sea (ICES). After completing international stock assessment exercises, ICES and STECF committees advise the EU Council of Ministers who decide annual ‘quotas’ i.e. Total Allowable Catches (TACs)^3^. TACs should be consistent with achieving maximum sustainable yield (MSY), but these have traditionally been ~20% higher than advised by scientific committees^4^. However, current reform of the CFP contains a commitment to manage fish stocks according to the best available scientific advice and to achieve MSY by 2020^5^.

Improved quality of fishery data including growth rates are needed for development of analytical assessment models and improvement of MSY reference points^6^. ‘Growth’ can be defined as the determination of body size as a function of age. Knowledge of mean length-at-age and associated growth descriptors is essential for the effective management of fish stocks. However, direct determination of age and growth in many crustaceans, including *N. norvegicus,* is unresolved due to a lack of verified aging criteria and the difficulties in measuring growth in moulting species. Previous growth studies on *N. norvegicus* have mainly employed indirect methods for estimating growth; for example, identifying cohorts within length frequency distributions and determining the rate of their progression over time (see Table 1). Numerical methods have also been developed which allow the conversion of length-frequency data into age composition, but the final interpretation of such approaches is only reliable when direct age readings are available to verify the age composition of length cohorts. These are not available in *N. norvegicus*. Other issues are the difficulties in distinguishing length-cohorts, as well as the high variability in the age-length relationship in crustaceans^7^. Finally, growth parameters generated from indirect methods generally rely on fisheries-derived data, hence are vulnerable to fisheries effects. Very few studies have been carried out on *N. norvegicus* from unexploited habitats^8^. Overall, without complementary biological information, the use of indirect length-frequency approaches produces unverifiable results^9^.

To counteract the above issues; growth can be measured directly, using tagging of wild crustaceans to determine somatic growth per unit time. Analysis of such data can reveal information about both increment at moult (i.e. the change in size over a moult) as well as frequency of moulting, which is important since smaller individuals may moult more frequently than larger ones per unit time^10^. Although tagging is a valuable tool to directly estimate growth increments, this is extremely costly and impractical, due to traditionally poor rates of tagged returns. In *N. norvegicus*, tagging studies have typically yielded small sample sizes. Hillis^11^ recovered only 23/957 individuals (2.4%) from a catch of 18,800 in the Irish Sea and Ulmestrand and Eggert^12^ reported 69/2106 recaptures (3.3%). Chapman^13^ reported better rates of recovery of up to 19.5% of released individuals within certain patches in west of Scotland, however he did not calculate any VBGF growth parameters for these individuals.

The present study investigated the growth of *N. norvegicus* on the Irish west coast via tagging with substantially increased recapture rates compared with most previous studies. The study population was located inshore, away from the trawling grounds and was generally lightly-exploited. To maximise tagged returns, the experimental area was left undisturbed by local creel fishermen (apart from scientific fishing) for >24 months of this study. Tagged returns provided size-specific growth averages and this information was used to generate growth descriptors for wild *N. norvegicus*, as well as to evaluate assumptions of a standard growth model (Von Bertalanffy Growth Function -VBGF). The frequency distribution of moult increments was used to estimate moult frequency in wild individuals and this parameter was also monitored in captivity. The importance of resolving uncertainties about growth is all the more critical since several functional management units (‘Functional Units’ -FUs) are overexploited; for example, *N. norvegicus* in FU15 (Western Irish Sea), FU17 (Aran Grounds), and FU19 (SE and SW Ireland), leading to separate management measures being proposed in these areas^6^,^14^. Finally, data from the present study makes a contribution towards examining important questions in marine fisheries including density-dependent growth depression, which has been suggested for *N. norvegicus* in certain circumstances^6^,^8^,^15^,^16^. The latter is a key issue because it has implications for resilience of stocks to fishing pressure.

## Results

### Evaluation of growth and moulting frequency

**Figure 1:**
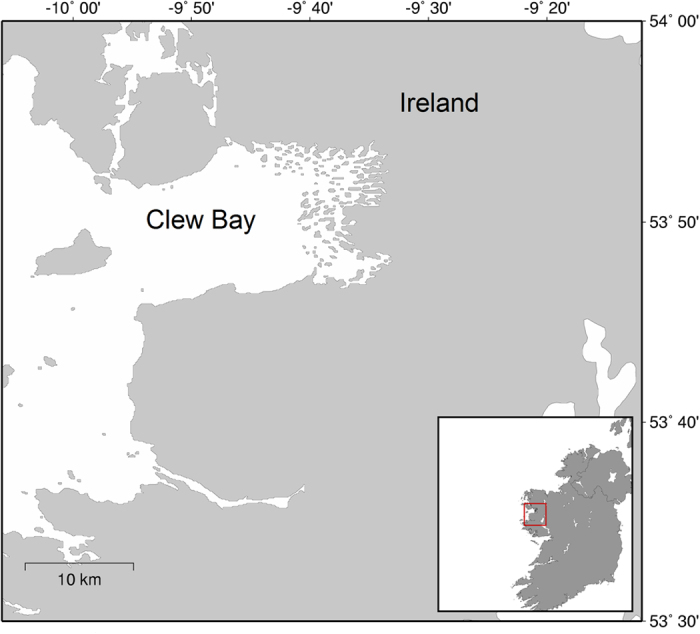
Inset: study area and release location of 1177 coded wire-tagged *N. norvegicus* in June and July 2013. 207 *N. norvegicus* (100 males and 107 females) were recaptured in April-September 2014 and a further 38 individuals were recaptured in June–July 2015 (12 males and 26 females). The authors acknowledge the use of the Maptool program for the production of this map. Maptool is a product of SEATURTLE.ORG (SEATURTLE.ORG Maptool. 2002, http://www.seaturtle.org/maptool/tos.shtml, accessed 08 February 2015).

207 tagged *N. norvegicus* were recaptured in year one at Clew Bay in the west of Ireland (Fig. 1), broken down into n = 100 males and n = 107 females. A further 38 tagged individuals were recovered in year two (n = 26 females and n = 12 males). The total rate of recaptures over both years was 245/1177 or 20.8%.

**Figure 2:**
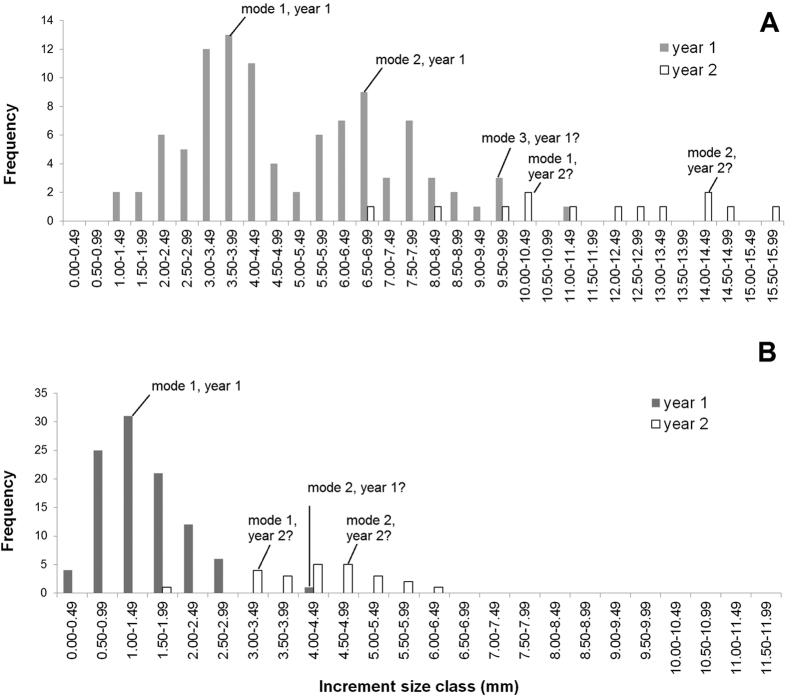
Evidence of bimodality i.e. two annual moults in male *N. norvegicus*. Frequency distribution of growth increments in (**A**) male and (**B**) female *N. norvegicus* in the west of Ireland; note different horizontal axes in (**A**,**B**). Individuals were at liberty for approximately one or two years and sample sizes were n = 99 (males year 1), n = 12 (males year 2), n = 100 (females year 1), n = 25 (females year 2).

**Figure 3:**
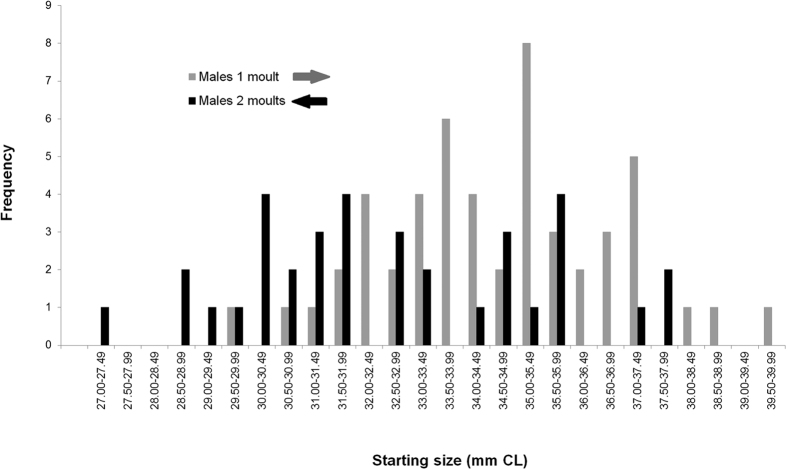
Moulting modality of males according to starting size. *N. norvegicus* which moulted once (i.e. had increments of <4.49 mm CL) or moulted twice (i.e. increments of 5.50–8.49 mm CL) are organised by starting size. The starting size distribution of males which moulted once is significantly larger (shifted to the right) relative to those which moulted twice –see Results. CL: Carapace Length.

Size increments indicated that most individuals grew during year one of the study, with n = 200/207 moulting at least once. The frequency distribution of size increments was bi-modal in males, indicating that two moults occurred in about half of the males during year one, with a possible third mode/moult occurring more rarely (Fig. 2A). The female distribution was more uni-modal (one moult) in year one, apart from a single individual who’s growth increment was large enough to indicate that it might have moulted twice (Fig. 2B). Limited samples sizes in year two prevented strong interpretation of this growth, however there was evidence of very wide-ranging growth increments, and hence multiple moults (Fig. 2A,B). Figure 3 examines whether starting size had an effect on moulting rate in males during year one (which was the only year with sufficient data to examine this). A significant t test (t = 3.69, df = 61, P < 0.0001) showed that the starting sizes of males which had moulted once (i.e. those with mode 1 increments <4.49 mm CL) was significantly larger than those which had moulted twice (i.e. mode 2 increments of 5.50–8.49 mm CL). Thus, smaller males moulted more frequently than larger ones.

**Figure 4:**
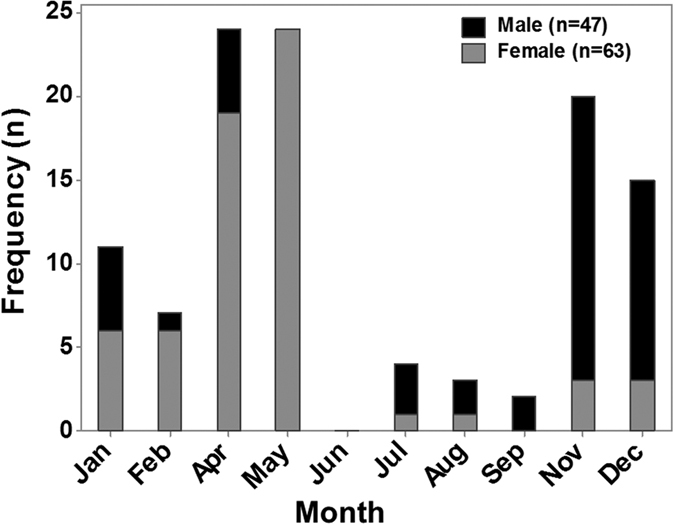
Frequency of moulting in male and female *N. norvegicus* during one year of captivity (n = 110). 47/111 males and 63/121 females moulted during this period. No individuals were observed to moult in June. Data were not recorded for the months of March and October.

**Figure 5:**
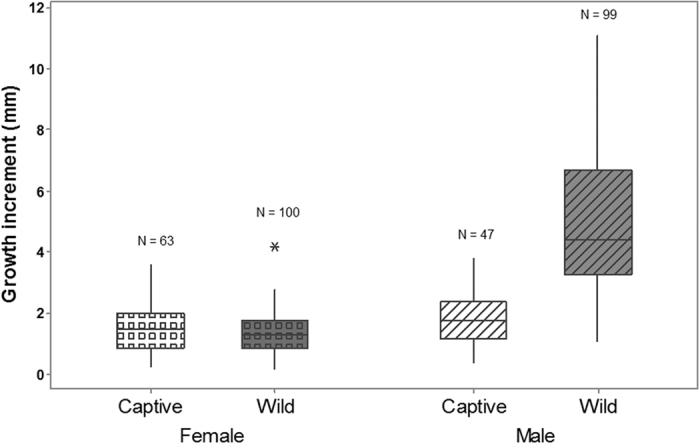
Growth increment variability between wild and captive male and female *N. norvegicus* in year one (2014; n = 309). The total range of increments exhibited by individuals in each group (perpendicular lines) and the median increment (horizontal lines) are shown.

In captivity, 47/111 males and 63/121 females moulted once and the remainder did not moult in a 12 months period. No individuals moulted more than once. Captive moulting occurred during most months, apart from June, however highest moulting was seen in April and May (females) or November and December (males) (Fig. 4). Growth increment was statistically lower in captive males (median = 1.00 (range 1.00–2.00) mm CL) compared with wild males (3.50 (0.40–11.10) mm CL; Mann-Whitney U = 301.0, n_wild_ = 99, n_captive_ = 47, P < 0.001) (Fig. 5). Growth was almost identical for wild and captive females, with no significant difference between these two groups (median_captive_ = 1.00 (1.00–2.00) mm CL, median_wild_ = 1.40 (0.20–4.20); Mann-Whitney U = 2732.5, n_wild_ = 100, n_captive_ = 63, P = 0.154) (Fig. 5).

**Figure 6:**
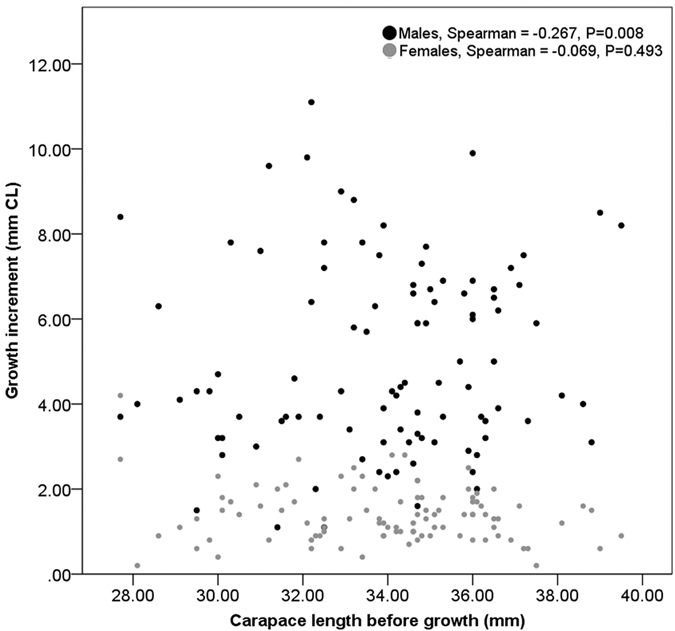
Carapace length before growth (i.e. starting size) versus growth increment for wild tagged male and female *N. norvegicus* captured in Clew Bay, Co. Mayo during year one (April–September; 2014). Larger males grew significantly less than smaller males. No trendline is provided, as this is a correlation. Female growth was not related to starting size.

Analysis of growth increments in the wild was broken down by year. In year one, the mean number of days that males were at liberty was 344 ± 3.4 standard error mean (S.E.), during which time mean growth was 5.1 ± 0.2 mm CL. Females were at liberty for an average of 344 ± 2.5 days, with mean growth of 1.4 ± 0.1 mm CL. Mean growth within size classes (Table 2) showed that smaller males had the highest growth increments, for example, growth of 7.3 mm CL was observed in a male in the 26–28 mm size class, compared with mean growth of only 5.3 (±1.6) mm in males of 38–40 mm. A significant negative correlation indicated lower growth in larger males relative to small ones, albeit these data had a large scatter (Spearman ρ = −0.267, p = 0.008). No such relationship was seen in females (Fig. 6). Apart from the smallest female size category, which grew by a mean of 3.5 ± 0.8 mm CL, female growth only varied slightly across size classes and averaged around 0.8–1.5 mm CL, irrespective of initial size (Table 2).

Substantial growth increments were seen in individuals which were at liberty for two years of growth i.e. 654 ± 6 days in males and 665 ± 6 days in females (Table 3). Largest increases over this period were seen in smaller males, up to 14.9 mm CL. Overall mean growth in males after two years was 12.0 (±0.8) mm CL. This compared with overall mean growth of 4.6 (±0.3) mm CL in females. A maximum increment of 6.18 mm CL was seen in one female individual. Like males, mean female growth after this period was highest in the smallest size class (5.5 ± 0.9 mm CL) (Table 3).

### Estimation of growth parameters

A total of 4785 *N. norvegicus* were captured between April and September 2014 by creel fishing (n = 1457 males; n = 3328 females). The overall modal size for the total catch was identical for males and females, whereas mean size was larger in males (40.2 ± 0.2 mm CL) than females (37.6 ± 0.1) (Supplementary Fig. S1). Monthly length-frequency distributions between April and September 2014 were plotted for all captured *N. norvegicus*, however no monthly progression of modes was evident for either sex (Supplementary Fig. S2). There was little evidence of recruitment of individuals <25 mm CL over the five months, apart from four females measuring between 19–22 mm CL in July (Supplementary Fig. S2).

**Figure 7:**
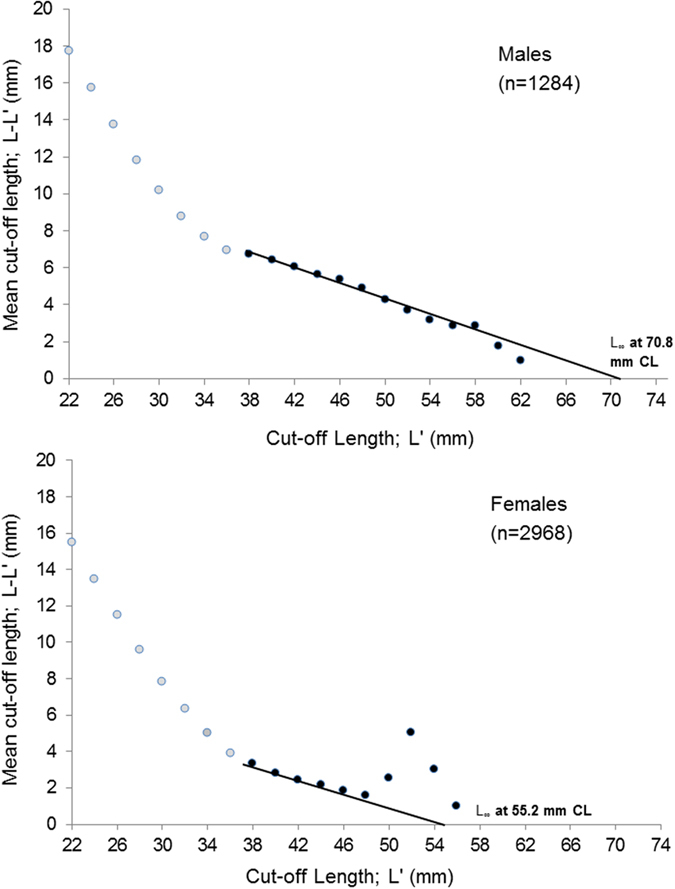
Powell-Wetherall plot with linear regression to determine L_∞_ for male (above) and female (below) *N. norvegicus* captured in Clew Bay, Co. Mayo (April–September; 2014). Regression analysis was carried out on the data points indicated in black, based on the minimum size that an individual reached before being ‘recruited’ to the fishery –see Methods.

L_∞_ was estimated separately in males and females using the Powell-Wetherall method for *N. norvegicus* captured between April and September 2014 (n = 4252, omitting tagged individuals). Based on the mean size distribution of individuals in Clew Bay, males were considered to be fully recruited to the fishery at a length of 38 mm CL and females also recruited at a length of 38 mm CL. The Powell–Wetherall analysis estimated asymptotic carapace lengths, L_∞_, in *N. norvegicus* to be 70.8 mm CL for males and 55.2 mm CL for females (Fig. 7). Using these estimates of L_∞_, “forced” Gulland and Holt plots were used to derive growth rate k from the tagged *N. norvegicus* that were recaptured in 2014. All tagged individuals showing zero or negative growth (n = 7) or unreadable tags (n = 1) were omitted from these plots, which resulted in an overall n = 99 males and n = 100 females. For males, k was estimated to be 0.157. For females this growth rate was considerably lower at 0.067. The Gulland and Holt plot calculations were repeated for year two growth (2015; n = 12 males and 25 females –a single individual was omitted from 2015 calculations due to a lost tag). Higher values of k were seen in 2015, i.e. 0.192 (males) and 0.117 (females). Pooling both years, k was estimated to be 0.161 in males and 0.077 in females at Clew Bay (Table 1).

## Discussion

Tag-recapture is the preferred method for estimating growth in exploited crustacean stocks because of many issues with indirect approaches involving analysis of modal (i.e. cohort) progression^17^,^18^,^19^. The present study illustrated the problem with the latter very well: cohorts were difficult to identify in *N. norvegicus* (Supplementary Fig. S1) and there was no progression of length modes across six months in >4000 captured individuals (Supplementary Fig. S2). Thus it was not possible to detect changes in growth by tracking modal sizes over this timeframe. ‘Flat’ population size structure in *N. norvegicus* is reasonably common^6^ and may be due to overlapping age or moulting groups, spatial patchiness in the size structure, poor representation of both small and large size classes in the sampled population, etc.^20^,^21^. This problem is exacerbated by a lack of verified aging criteria because length cohorts cannot be examined directly for the purposes of separating these into component age groups.

On the other hand, tag-recapture is difficult for more practical reasons because tagging can result in issues arising from repeat handling of animals, interference with the normal range of movements or moulting, and this type of study has traditionally shown a low return rate of tagged individuals (e.g. refs 11 and 12). But a parallel study has shown that tag retention was high and there were no negative effects associated with the tagging procedure on moulting frequency, growth increment or mortality in *N. norvegicus* (unpublished data). In addition, the present study was carried out in inshore areas, where agreements were made with local fishermen to allow the release area to remain ‘fallow’ over the experimental period. Local fishermen were eventually compensated as they were chartered to recover tagged *N. norvegicus* in their creels the following year. As a result, the recapture rate of tagged *N. norvegicus* was relatively high in the current study (20.8%, n = 245/1177). Most *N. norvegicus* were recaptured after approximately one year of growth, with a good balance of returns across males (n = 100) and females (n = 107) to provide robust growth estimates for both sexes.

It was apparent that mean annual growth increments were much larger in males (5.1 ± 0.2 mm CL) than in females (1.4 ± 0.1 mm CL). In females, growth increment was reasonably uniform within and across size classes apart from the smallest size class (<28 mm CL), which had a much bigger increment (Fig. 6 and Table 2). The transition to sexual maturity occurs in 50% of the Scottish stock at approximately 26–29 mm CL^22^, so this growth slow-down in females is likely to be associated with the onset of sexual maturity^23^. Meanwhile, growth increments were statistically higher, on average, in smaller males than in larger ones (Fig. 6; Table 2). This is important for growth prediction because an assumption of the VBGF is that the rate of growth over time slows down in larger individuals^23^. Additional *N. norvegicus* individuals were recaptured after approximately two years of growth (n = 38). Although the relatively low numbers of returns in this case means that these data are likely to suffer from small sample artefacts, again we observed that the smallest size classes of males and females showed the largest growth increments after two years of growth. Overall, the growth of *Nephrops* appeared to conform to assumptions that permit use of the VBGF^24^. Reports in the literature on the growth of *N. norvegicus* are somewhat contradictory. Although, like us, others have found decreased growth increment with increased starting size^25^,^26^, a positive correlation between starting size and increment was reported in lab-grown males by González-Gurriarán *et al.*^19^,^20^ and in wild males by Charuau^27^ and Chapman^13^. Using limited size ranges, different analytical methods, or too few individuals might explain contradictory findings^9^, but so too might challenges in sampling growth properly in this species.

The second major element of growth is the question of moulting frequency. The observed changes in size demonstrated that annual moulting occurred in almost all wild individuals over the size range examined (26–40 mm CL). Zero or negative growth was rarely observed in this group. In fact, almost half of the males moulted twice (Fig. 2), which helps to quantify the largely unresolved issue of moulting frequency of *N. norvegicus* at this latitude^13^,^28^. Up until now, mature males have been considered to moult ‘at least once’ in the north Atlantic, with mainly circumstantial information available about how many males actually do this. We also observed that more frequent moulting was associated with smaller starting sizes (again, conforming to the VBGF assumption, since this assumes small males grow more). However, size is probably only one of several factors which promote higher moulting frequency. The rate of moulting and growth depends on a suite of factors all of which likely act in tandem and are therefore difficult to separate in the wild^20^,^29^,^30^,^31^. Access to food and territorial interactions are important factors that influence the growth of individuals^32^,^33^. These factors may co-vary with starting size, so that the smaller individuals may have more physiological scope for growth (in accordance with the VBGF assumption), but less access to the resources they require to do so (e.g., because they may suffer negative territorial interactions with larger individuals).

Both moulting frequency and increment at moult were much reduced in captive individuals, particularly in males. Reduced growth in captivity has been shown in numerous studies^9^,^20^, highlighting the value of wild growth estimates. There was a seasonal signal in moulting of captive *N. norvegicus* females (April and May). Moulting in wild females also occurs in April and May, since this is the period immediately following spawning of their embryo masses at Clew Bay (approximately third week of April; unpublished data). Females not bearing eggs are free to moult outside of this period, however. Moulting in captive males mainly occurred in November and December, but was not confined to this period. The majority of male moulting in the wild appears to occur over an extended period from Spring-Autumn^34^.

Even minor changes in values of VBGF parameters (asymptotic length L_∞_ or growth rate k) lead to drastic changes in the biological reference points for fisheries. Depending on which growth parameters are used, the management advice could either advocate increased or reduced fishing effort. It is recommended to calculate growth rates (k) independently of L_∞_, as in the present study^12^. L_∞_ for males in Clew Bay, Co. Mayo in the west of Ireland was 70.8 mm CL. Since the largest male encountered during an independent fishing study at Clew Bay in 2015 was 68.0 mm CL, i.e. less than theoretical maximum L_∞_, this provides confidence in our L_∞_ estimation. This value was similar to an estimate of 72.9 mm CL from the Skagerrak^12^ (Table 1), with both studies using similar approaches. Meanwhile, the male growth rate, k was higher in Ireland than in the Skagerrak (0.161 compared to 0.138). Female asymptotic growth in the current study was 55.2 mm CL, which we believe to be an underestimate, since the largest female captured in the field at Clew Bay was 57.0 mm. Both values are substantially lower than maximum female size in the Skaggerak (64.9 mm CL); but, as with the males, the growth rate of females was higher in Ireland.

A key question in fisheries management is that of productivity across management areas. Densities on fishing grounds, population size distributions and growth rates give an indication of the degree of fishing pressure which may be applied. For example, we can compare *N. norvegicus* stocks on the east coast (Irish Sea) which occur at high-densities and lower-density stocks on the west coast^8^. High densities in the Irish Sea are suggested to depress mean somatic size, and indeed, the L_∞_ for east coast males was 18% lower (60 mm CL) compared to the west (see also refs 8, 15 and 16). Meanwhile, L_∞_ in east and west coast females was similar (respectively 56.0 versus 55.2 mm CL –Table 1), but this time, the growth rate of females in the east coast (k = 0.100) was substantially higher than in the west (k = 0.077). If high densities are supressing body size in Irish Sea males and female growth rates are higher, this stock may be more resilient to heavier fishing. Conducting a tagging study to directly compare growth as a function of density would definitively resolve this question since current growth estimates in the Irish Sea are indirect. Growth rates may be up to three times higher (males) or seven times higher (females) in different stocks across the range of *N. norvegicus*, with a tendency towards higher growth at the southern end of the species range (Table 1). While some of this variability undoubtedly arises due to genuine differences in growth, experimental artefacts are also very likely because of the range of techniques used to estimate growth.

Finally, growth rates have been expressed in both lengths and weights (Tables 2 and 3), since the increase in biomass which occurred while tagged individuals were in the wild can inform inshore fishers on economic returns from local grounds, allowing a cost-benefit analysis of re-stocking or ‘ranching’ activities to be carried out. For example, all n = 199 recaptured tagged *N. norvegicus* increased in total weight by 2.09 kg when allowed to grow for 344 days (on average) in inshore grounds. The average weight of a single individual tagged in 2013 was 0.029 kg, so that 35 individuals produced ~1 kg of whole product. In 2014, the average weight of a single tagged *N. norvegicus* increased to 0.039 kg, with only 25 individuals required to produce ~1 kg of product. Therefore the average increase in weight per individual was equivalent to progression into a new size-grading category^35^ prior to sale. This quantifies the economic benefit of ‘ranching’ *N. norvegicus* in inshore areas and potentially seeding with stock from trawling grounds. Growth in the wild after two years was at a slightly higher rate than after one year (12.0 mm CL in males, or 4.6 mm CL in females, on average -Table 3), which gives further insight into rapid growth in this species.

## Materials and Methods

### Tag-recapture experiment

All experimental animals were captured in Clew Bay (-see below) during April 2014 using static gear (creels or ‘pots’). Creels were baited with salted herring and soaked for 48 hours before being lifted and examined for *N. norvegicus* in a range of sizes (see below). Although emergence behaviours along diel, tidal and other rhythms strongly influence catchability of *N. norvegicus* in trawling gear^36^,^37^,^38^, these effects are much less likely with static gear which samples over an integrated period. For transport to the aquaculture facility, animals were housed in a damp, chilled fisherman’s cassette, which is a device normally used for storing *N. norvegicus* during fishing operations. Once arrived at the aquaculture facility, animals were housed in individual plastic tubes, which were stacked by attaching the tubes to a vertical support within large tanks (5 m^3^) in a flow-through aquaculture system (flow rate of 50–65 m^3^/hour and an average monthly temperature 10–17 °C). *N. norvegicus* in the system were fed once a week with frozen mussel until the time came to use them in the tag-recapture growth experiment.

Tagging was carried out using sequential CWTs (Northwest Marine Technology Inc.), which are passive tags that have been successfully employed to tag a range of crustaceans^39^,^40^,^41^. Made of stainless steel, these tags come in various sizes, but the smallest tag (1.1 mm length, 0.25 mm in diameter) was chosen for the present study. The tags were loaded, one at a time, into a syringe for injection into the ventral musculature of the 3^rd^ abdominal segment of *N. norvegicus* (numbering segments from the posterior of the animal). At recapture, it is necessary to dissect out these tags in order to read the unique sequence under the microscope, thereby identifying individuals. Animals were allowed several days to recover from the tagging procedure prior to release onto fishing grounds.

Tagged animals were released back to their original capture location in Clew Bay, which is a broad bay on the west coast of Ireland that faces the Atlantic Ocean (Fig. 1). The bay is approximately 25 km long and 12.5 km wide, covering an area of approximately 31,259 ha^42^. Much of this bay contains glacially-formed drumlins, which extend westwards some 10 km into the body of the bay^43^. The varied topography creates a complicated current regime and bathymetry, as relatively deep channels often surround the many islands of the inner bay and water depths can vary over relatively short distances. The substrate at the release location was mainly medium to fine sand according to Folk particle size categories (unpublished data). Scientific divers confirmed that the habitat was suitable and there was a resident population of *N. norvegicus* at the release site.

A total of 1177 *N. norvegicus* were tagged and released at Clew Bay on three dates in 2013: 5 June, 19 June and 17 July. The releases were conducted at 18–20 m depth, depending on the state of the tide, which varies ~5 m in vertical extent. Tagged individuals were released at three closely-located sites, approximately 30 m apart. The size of individuals varied between 26.9–40.2 mm CL in males and 22.0–44.6 mm CL in females. Tagged individuals were released to the seafloor in a fisherman’s cassette that was placed in a weighted device that sat on the seabed for ~72 hours, allowing individuals to escape at will. Creel fishing for *N. norvegicus* at Clew Bay is seasonally restricted to Spring and Summer months, but in the present case, the release location was left ‘fallow’ to fishing activity post-release. This ‘closed’ experimental period handled the problem of short recapture times and growth over-estimation, which can occur in animals with periodic growth (moulting) when growth is measured over too short a time period. Creel fishing resumed from April to September 2014 (i.e. approximately one year post-release) and again in June–July 2015 (approximately two years later). Scientific observers were on board during all fishing operations to measure the entire catch (mm CL) and to recapture as many of the tagged *N. norvegicus* as possible. Tagged individuals were identified due to the steel tag emitting a ‘beep’ after passing animals over a T-wand detector (Northwest Marine Technology Inc.). All tagged individuals were returned to the laboratory for measurement (mm CL) and dissection of CWTs for reading under the microscope.

### Evaluation of growth and moulting frequency

A frequency distribution of the increment (i.e. change in size in mm CL) while individuals were at liberty was generated separately in males and females to investigate the presence of modes in the distribution, and hence, the number of moults. As the moulting frequency cannot be measured directly in the wild, moulting frequency was also observed in captive *N. norvegicus*. Experimental animals were caught and held in an aquaculture system, as outlined above, and moulting frequency was observed over 12 months in April 2014–March 2015. A total of 232 *N. norvegicus* were held over size ranges of 26.2–53.0 mm CL for males and 23.5–41.2 mm CL for females. CL measurement was carried out approximately once every four weeks, however no observations were made in two of the months (March and October). Regular checks determined whether *N. norvegicus* within the holding system had moulted. Moulting was detected either by the presence of shell fragments from the moulted exoskeleton, by a change in the appearance of the individual e.g. a pink/soft shell, or by a sudden change in CL (defined as an increase of >0.5 mm which was sustained over subsequent months).

Growth increment expressed in both CL and weight (g) was tabulated according to starting size (i.e. size at tagging) in wild *N. norvegicus*. Spearman rank correlation was used to examine a statistical relationship between CL before growth and growth increment. Comparison of growth increment after year one was carried out in wild and captive *N. norvegicus* using a Mann-Whitney U test. These statistical analyses were carried out using MINITAB 17 Statistical Software Package.

### Estimation of growth parameters

Two standard growth parameters were estimated: L_∞,_ which is the asymptotic length i.e. the length that an individual would reach if it was allowed to grow indefinitely; and k, which is the rate at which L_∞_ is approached. These quantities are commonly used in growth models including the VBGF^24^. Growth in Crustacea can be described using VBGF since the stepped growth in moulting species can average out as a smooth curve when viewed for an entire cohort of individuals. However, the assumptions of VBGF should still be met (see below). Separate analyses were chosen to generate L_∞_ and k because of their strong inter-dependence (one quantity implies the other). L_∞_ was calculated from analysis of length-at-catch distributions obtained during 2014 fishing activity while k was calculated from growth increments obtained from the tag-recapture experiment. The Powell–Wetherall method can be used to obtain an estimate of L_∞_ using catch data by estimating length classes equal to and above the fully recruited or ‘cut-off’ length. This method uses linear regression of terms in equation (1)^44^,^45^,^46^:


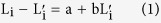


A mean length sample L_i_ above a cut-off length L_i_*′* is defined, where L_i_*′* represents the smallest fully recruited individuals for each size class i and L_i_ is the mean length for all individuals above L_i_*′*. There is generally a linear relationship between corresponding values of L_i_*′* and L_i_, so ‘a’ in this equation represents the regression constant (intercept), which corresponds to L_∞_ and ‘b’ the regression coefficient (slope).

The assumptions of this method are that growth follows the VBGF, i.e. there is a linear decline in growth with increased length. The sample population is also assumed to be in equilibrium (at a steady-state) with constant exponential mortality, no changes in selection pattern of the fishery and constant recruitment^47^.

‘Forced’ Gulland and Holt plots were used to estimate the growth rates k, with data from recaptured tagged *N. norvegicus.* A Gulland and Holt plot calculates k based on the length of individuals when they are first measured, the length of individuals when they are measured again, and the difference between these two measurements per unit time. The regression was “forced” using L_∞_ calculated in the previous analysis, allowing k to be estimated separate from L_∞_ –see ref. 12. Powell-Wetherall and Gulland and Holt plots, as well as all statistical regressions related to these plots were carried out using the fisheries software program, FISAT II^48^. A final estimate of k was provided by pooling the appropriate tagged returns for 2014 and 2015 (n = 236).

All animal sampling was carried out in accordance with relevant guidelines and regulations from the relevant authority (Sea Fisheries Protection Agency, Ireland). Animals were held in a licensed aquaculture facility. Animal handling and experimental procedures were carried out under supervision of certified personnel (Laboratory Animal Science & Training Certification), which is in keeping with University Policy.

## Additional Information

**How to cite this article**: Haynes, P. S. *et al.* Growth in *Nephrops norvegicus* from a tag-recapture experiment. *Sci. Rep.* **6**, 35143; doi: 10.1038/srep35143 (2016).

## Supplementary Material

Supplementary Information

## Figures and Tables

**Table 1:** A comparison of growth parameters for *Nephrops norvegicus* across Atlantic areas.

Location	Males		Females		Method	Reference
	k (yr^−1^)	L_∞_ (mm CL)	k (yr^−1^)	L_∞_ (mm CL)		
Skagerrak	0.138	72.9	0.056	64.9	Powell-Wetherall/Gulland & Holt, Tag-recapture	12
Scottish West (Jura)	0.166	57.0	0.228	46.6	Petersen’s method (modal progression), & tag-recapture	15
Scottish West (Clyde)	0.160	73.2	0.156	68.9		
Irish Sea (east coast)	0.160	60.0	0.100	56.0	Unknown	49
Irish west coast	0.161	70.8	0.077	55.2	Powell-Wetherall/Gulland & Holt, Tag-recapture	Present study
Portugal (northern)	0.380	88.3	0.380	64.5	Gulland & Holt, modal progression (ELEPHAN)	50
Portugal (central)	0.410	94.9	0.440	69.2		
Portugal (south)	0.140	78.9	0.120	71.3	Gauss-Newton FISHPARM	21
Spain (Atlantic)	0.130	83.4	0.120	70.7	Length-Cohort Analysis	51

**Table 2:** Mean ± Standard Error of mean (S.E.) growth increments for wild tagged *Nephrops norvegicus* after n = 344 ± 3.4 days (males) or 344 ± 2.5 days (females) across a range of size classes.

Size class/mm CL	Males Number recaptured	Mean growth increment/mm CL	Mean growth increment/mm TL	Mean increase in weight/g	Females Number recaptured	Mean growth increment/mm CL	Mean growth increment/mm TL	Mean increase in weight/g
26–28	1	7.3 (±0.0)	24.2 (±0.0)	16.2 (±0.0)	2	3.5 (±0.8)	11.4 (±2.5)	7.0 (±1.7)
28–30	5	6.5 (±0.7)	21.6 (±2.5)	16.6 (±2.2)	7	0.8 (±0.1)	2.7 (±0.4)	1.7 (±0.3)
30–32	18	6.0 (±0.4)	19.9 (±1.4)	17.3 (±1.4)	14	1.7 (±0.2)	5.6 (±0.5)	4.1 (±0.4)
32–34	27	4.7 (±0.5)	15.7 (±1.5)	15.1 (±1.7)	22	1.3 (±0.1)	4.3 (±0.4)	3.6 (±0.4)
34–36	30	4.9 (±0.4)	16.1 (±1.4)	17.9 (±1.9)	30	1.5 (±0.1)	4.9 (±0.4)	4.6 (±0.4)
36–38	14	4.1 (±0.4)	13.7 (±1.3)	16.5 (±1.9)	20	1.2 (±0.1)	4.1 (±0.4)	4.3 (±0.4)
38–40	4	5.3 (±1.6)	17.5 (±5.2)	24.1 (±8.2)	5	1.2 (±0.2)	4.4 (±0.6)	4.6 (±0.7)
Average		5.1 (±0.2)				1.4 (±0.1)		

A conversion factor^52^ was used to convert size in carapace length (CL) into total body length (TL).

**Table 3:** Mean ± Standard Error of mean (S.E.) growth increments for wild tagged *Nephrops norvegicus* after n = 654 ± 6 days (males) or 665 ± 6 days (females) across a range of size classes.

Size class/mm CL	Males Number recaptured	Mean growth increment/mm CL	Mean increase in weight/g	Females Number recaptured	Mean growth increment/mm CL	Mean increase in weight/g
26–28	0	n/a	n/a	0	n/a	n/a
28–30	1	14.9 (±0.0)	(47.3 ± 0.0)	1	5.5 (±0.9)	(14.6 ± 0.0)
30–32	3	13.2 (±0.5)	(49.2 ± 2.6)	9	4.9 (±0.6)	(13.1 ± 2.6)
32–34	2	9.1 (±2.3)	(27.4 ± 2.9)	5	3.7 (±0.7)	(11.2 ± 2.3)
34–36	3	12.8 (±1.6)	(49.6 ± 4.8)	7	4.8 (±0.4)	(15.7 ± 2.5)
36–38	3	10.9 (±1.7)	(46.5 ± 9.8)	3	4.2 (±0.1)	(11.3 ± 1.0)
38–40	0	n/a	n/a	0	n/a	n/a
Average		12.0 (±0.8)			4.6 (±0.3)	
