# Supplementary material for: Growth in Nephrops norvegicus from a tag-recapture experiment
Source: Sci Rep. 2016 Oct 11;6:35143. doi: 10.1038/srep35143 (PMC5057119; doi:10.1038/srep35143)
Supplement: Supplementary Information [file srep35143-s1.doc]

**Comprehensive evaluation of growth in *Nephrops norvegicus* using tag-recapture**

Paula S. Haynes, Patricia Browne, Liam Fullbrook, Conor T. Graham, Lee Hancox, Mark P. Johnson, Valentina Lauria, Anne Marie Power

Supplementary Figure S1


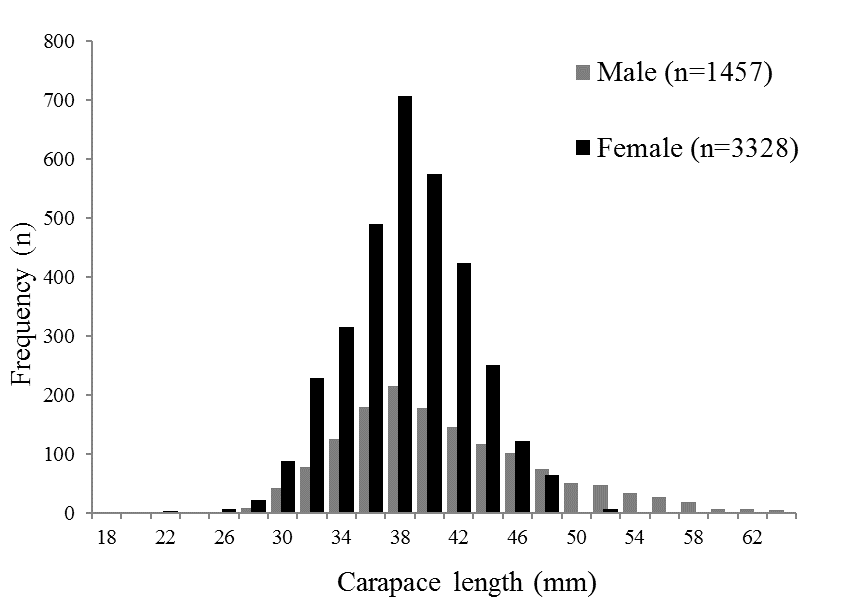


Supplementary Figure S1: Size distribution of all male and female *N. norvegicus* captured by creel fishing in April – September 2014 in Clew Bay Co. Mayo (n = 4785). It is common for size distributions to be similar across the sexes at this time of year, since mature females are represented in the fishery during this time.

Supplementary Figure S2


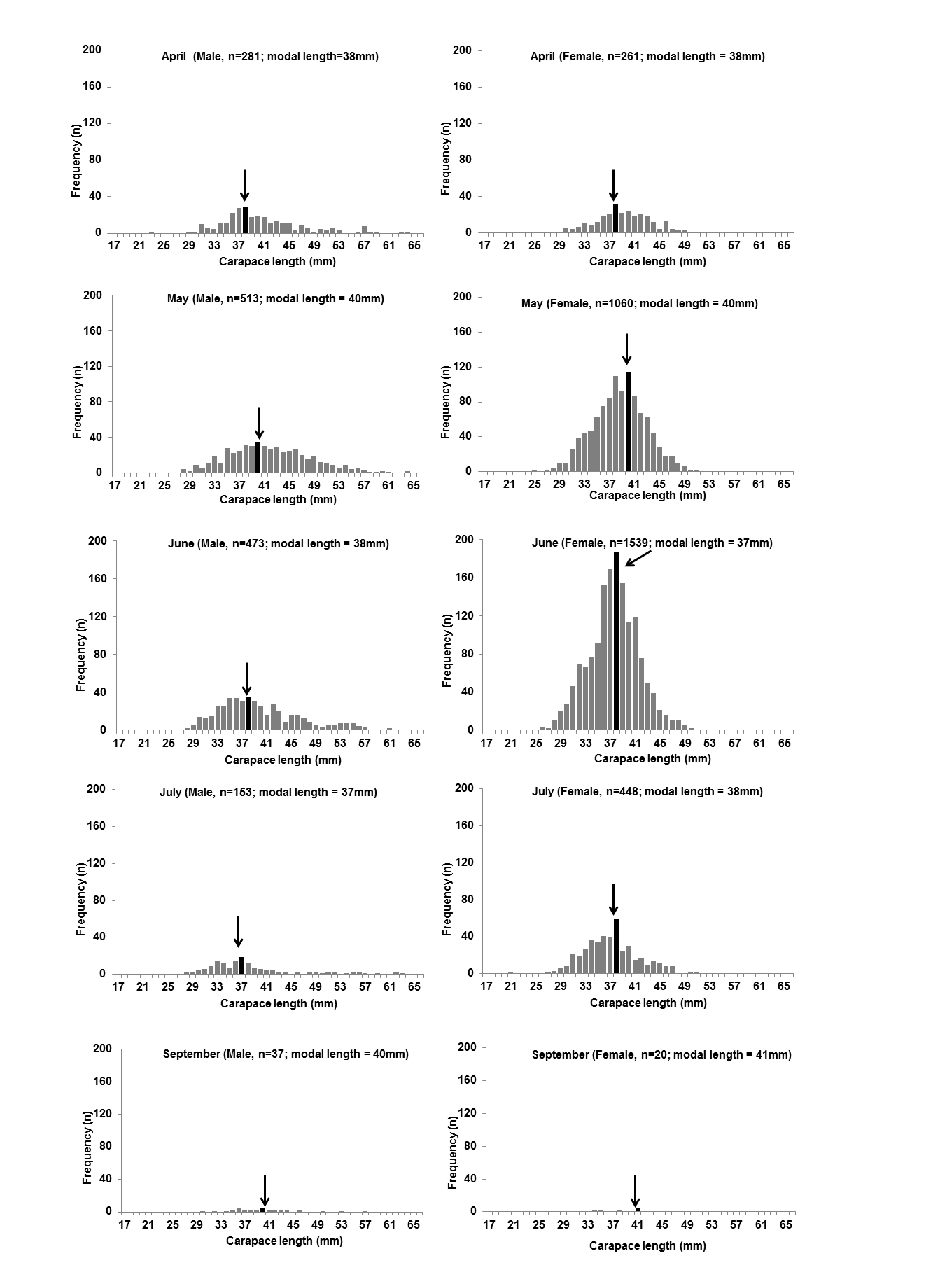
Supplementary Figure S2: Monthly length-frequency distributions of male (left) and female (right) *N. norvegicus* in Clew Bay, Co. Mayo in April – September 2014. The modal length each month is highlighted using black arrows.
